# The Essentials of Protein Import in the Degenerate Mitochondrion of *Entamoeba histolytica*

**DOI:** 10.1371/journal.ppat.1000812

**Published:** 2010-03-19

**Authors:** Pavel Dolezal, Michael J. Dagley, Maya Kono, Peter Wolynec, Vladimir A. Likić, Jung Hock Foo, Miroslava Sedinová, Jan Tachezy, Anna Bachmann, Iris Bruchhaus, Trevor Lithgow

**Affiliations:** 1 Department of Biochemistry and Molecular Biology, Monash University, Clayton Campus, Melbourne, Victoria, Australia; 2 Bio21 Institute, University of Melbourne, Parkville, Victoria, Australia; 3 Department of Parasitology, Faculty of Science, Charles University, Prague, Czech Republic; 4 Bernhard Nocht Institute for Tropical Medicine, Hamburg, Germany; University of Geneva, Switzerland

## Abstract

Several essential biochemical processes are situated in mitochondria. The metabolic transformation of mitochondria in distinct lineages of eukaryotes created proteomes ranging from thousands of proteins to what appear to be a much simpler scenario. In the case of *Entamoeba histolytica*, tiny mitochondria known as mitosomes have undergone extreme reduction. Only recently a single complete metabolic pathway of sulfate activation has been identified in these organelles. The *E. histolytica* mitosomes do not produce ATP needed for the sulfate activation pathway and for three molecular chaperones, Cpn60, Cpn10 and mtHsp70. The already characterized ADP/ATP carrier would thus be essential to provide cytosolic ATP for these processes, but how the equilibrium of inorganic phosphate could be maintained was unknown. Finally, how the mitosomal proteins are translocated to the mitosomes had remained unclear. We used a hidden Markov model (HMM) based search of the *E. histolytica* genome sequence to discover candidate (i) mitosomal phosphate carrier complementing the activity of the ADP/ATP carrier and (ii) membrane-located components of the protein import machinery that includes the outer membrane translocation channel Tom40 and membrane assembly protein Sam50. Using *in vitro* and *in vivo* systems we show that *E. histolytica* contains a minimalist set up of the core import components in order to accommodate a handful of mitosomal proteins. The anaerobic and parasitic lifestyle of *E. histolytica* has produced one of the simplest known mitochondrial compartments of all eukaryotes. Comparisons with mitochondria of another amoeba, *Dictystelium discoideum*, emphasize just how dramatic the reduction of the protein import apparatus was after the loss of archetypal mitochondrial functions in the mitosomes of *E. histolytica*.

## Author Summary

All eukaryotic organisms have mitochondria, organelles cordoned by a double membrane, which are descendants of an ancestral bacterial endosymbiont. Nowadays, mitochondria are fully integrated into the context of diverse cellular processes and serve in providing energy, iron-containing prosthetic groups and some of the cellular building blocks like lipids and amino acids. In multi-cellular organisms, mitochondria play an additional vital role in cell signaling pathways and programmed cell death. In some unicellular eukaryotes which inhabit oxygen poor environments, intriguing mitochondrial adaptations have taken place resulting in the creation of specialized compartments known as mitosomes and hydrogenosomes. Several important human pathogens like *Entamoeba histolytica*, *Giardia intestinalis*, *Trichomonas vaginalis* and microsporidia contain these organelles and in many cases the function and biogenesis of these organelles remain unknown. In this paper, we investigated the protein import pathways into the mitosomes of *E. histolytica*, which represent one of the simplest mitochondria-related compartment discovered yet. In accordance with the limited organellar proteome, we show that only core components of mitochondria-related protein import machines are present in *E. histolytica* to serve for the import of a small set of substrate proteins.

## Introduction

Mitosomes and hydrogenosomes are metabolically-specialized forms of mitochondria, found in some of the unicellular pathogens which inhabit oxygen poor environments [1]. A lack of a recognizable mitochondrial compartment had led to the proposal of a group of primitive, primarily amitochondriate, eukaryotes [2]. However, recent evidence has shown the organelles referred to as hydrogenosomes and mitosomes in the ‘amitochondriate eukaryotes’ to be highly evolved mitochondria, having reduced their metabolic pathways as a response to their anaerobic and partly parasitic lifestyles in diverse eukaryotic lineages [1], [3]–[6]. There is no eukaryote known to be primarily amitochondriate, and even secondary loss of mitochondria has not been found.

The biogenesis of mitochondria is the defining aspect of the organelle and depends on the import of proteins from the cytosol, driven by a set of characteristic protein translocases installed in the outer and inner mitochondrial membranes. Mitochondrial precursor proteins are translated on ribosomes in the cytosol, and then recognized by a protein *t*ranslocase in the *o*uter *m*itochondrial membrane (the TOM complex). This TOM complex imports precursor proteins through a channel formed by the essential subunit Tom40. Subsequently, imported proteins are transferred to the *s*orting and *a*ssembly *m*achinery (SAM complex) for assembly into the outer membrane, or one of two *t*ranslocases in the *i*nner *m*itochondrial membrane: the TIM22 complex for assembly into the inner membrane, or the TIM23 complex for translocation through the membrane and into the mitochondrial matrix [7],[8]. The presence of components of the TOM, TIM and SAM complexes in hydrogenosomes and mitosomes shows these organelles to be mitochondria, despite the impressive metabolic simplification that have taken place in these specialized compartments [9]–[13].

Mitosomes are the simplest form of mitochondria: they have lost their capacity for ATP synthesis, lost all vestiges of a mitochondrial genome and so far only limited set of proteins have been localized into these tiny double membrane-bound vesicles. This secondary reduction of function has occurred independently in distinct lineages of eukaryotes, being well characterized in the diplomonad *Giardia intestinalis* [14], the microsporidians (such as *Encephalitozoon cuniculi* [15], *Antonospora locustae* [12], *Trachipleistophora hominis* [16]) and the amoebozoan *Entamoeba histolytica* [17],[18]. The majority of known proteins found in the mitosomes of *G. intestinalis* and microsporidia are functional counterparts of mitochondrial proteins found in other organisms, and a unifying feature of all these organelles is their role in the synthesis of iron-sulfur clusters [14],[19],[20]. So far it is the sole metabolic process known to occur in mitosomes of *G. intestinalis* and microsporidia and conflicting data exist on the presence of the iron-sulfur clusters biosynthesis in *E. histolytica* mitosomes [21],[22]. In addition to being widespread in hydrogenosomes and mitosomes, the biogenesis of iron-sulfur centers is the only essential metabolic role of mitochondria in the model organism *Saccharomyces cerevisiae* [23].

*Entamoeba histolytica*, the causative agent of invasive amoebiasis in humans, seems to have taken further steps towards the extreme reduction of the mitochondrial compartment [24]. It represents the only known eukaryote in which the synthesis of iron-sulfur clusters is mediated by an NIF (nitrogen fixation) system acquired by horizontal gene transfer from an ε-proteobacterium [25]. According to prediction algorithms this biosynthetic pathway is predicted to be present in the cytosol instead of the mitochondrial compartment [26]. Consistently, Mi-Ichi et al, did not find either of Nif proteins in their mitosomal proteomic analysis [22] and also no iron-sulfur cluster containing protein is known to be present in the organelles as a candidate substrate for the NIF system [21]. However, Maralikova et al, presented data arguing for the dual localization of both Nif proteins with their specific enrichment in the mitosomes [21].

So far, the mitosomes of *E. histolytica* represent one of the simplest mitochondria known. With the presence of sulfate activation pathway in the mitosome the need for ATP in addition to the molecular chaperones within the organelle is obvious [17], [27]–[29]. Although the ADP/ATP carrier in the mitosomal membrane provides for ATP import [22],[27], two questions are left open. Firstly, how do mitosomes recycle inorganic phosphate (P_i_) arising from ATP hydrolysis? Secondly, how are all the mitosomal proteins transported across the membranes of the organelle?

To address these questions, we performed hidden Markov model (HMM) searches of the *E. histolytica* genome and discovered candidate sequences for (i) a P_i_ carrier to complement the activity of the ATP/ADP carrier, (ii) Tom40, a channel for substrate protein transport across the outer mitosomal membrane, and (iii) Sam50, an assembly machine for Tom40 in the outer membrane. The mitosomal protein import pathway in *E. histolytica* is mitochondrial in nature. Analysis of the mitochondrial protein import machinery of the related amoeba *Dictyostelium discoideum*, suggests that *E. histolytica* has extensively stripped the mitochondrial protein import machines to their essentials. This remarkable degeneration of protein import is in keeping with the apparent paucity of proteins imported into mitosomes in *E. histolytica*.

## Results

### Substrate transport by mitochondrial carrier proteins in *E. histolytica*

The family of mitochondrial carrier proteins is well characterized in terms of primary sequence motives and crystal structure. According to the structure of the bovine ADP/ATP carrier, six transmembrane segments form an α-helical bundle, consisting of three homologous modules [30]. Each of these modules contains a signature motif in the odd-numbered helices [P]-x-[D/E]-x-x-[K/R]. The proline residue in the motif introduces a kink into three of the six transmembrane helices. In order to obtain a sensitive tool for analysis of *E. histolytica* genome, a hidden Markov model was built that describes the defining features of the mitochondrial carrier protein family.

**Figure 1:**
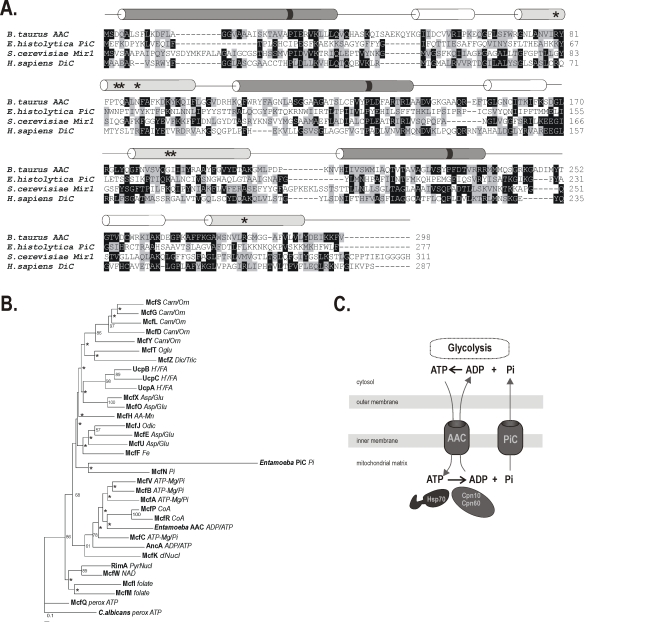
Mitochondrial phosphate carrier in *E. histolytica*. (**A**) Protein sequence alignment of *Eh*PiC with *Bos taurus* ADP/ATP carrier, *S. cerevisiae* phosphate carrier and human dicarboxylate carrier. The secondary structure is schematically depicted by colored cylinders above the alignment according to [30]. The odd- and even-numbered transmembrane α-helices are shown in dark or light grey, respectively. The short helices exposed to the mitochondrial matrix are shown in white. The black stripes on the helices depict the presence/absence of conserved proline residues in the mitochondrial carriers signature motif. Asterisks indicate substrate contact sites. (**B**) Protein maximum likelihood tree of 34 mitochondrial carrier proteins from *D. discoideum* together with the ADP/ATP carrier and PiC carrier from *E. histolytica* constructed by Phyml [80]. *C. albicans* peroxisomal ADP/ATP carrier was used as the outgroup. The putative substrates of the carriers in *D. discoideum* are inferred from sequence similarity to *S. cerevisiae* and human carrier proteins [31] and indicated as follows: *Carn/Orn* – carnitine or ornithine, *Asp/Glu* – aspartate/glutamate, *Oglu* – 2-oxoglutarate, *Dic/Tric* – dicarboxylate/tricarboxylate, *Pi* – phosphate, *PyrNucl* – pyrimidine NTP/NMP, *perox ATP* – peroxisomal ATP carrier, *H^+^ FA* – H^+^ fatty acid, *CoA* – coenzyme A, *dNucl* – deoxynucleotide, *AA-Mn* – amino acid (Mn^2+^), *Fe* – iron (mitoferrin), asterisks denote nodes with boostrap value lower than 50. (**C**) A schematic representation of *Eh*PiC and *Eh*ADP/ATP carrier in the mitosomes, depicting the role of *Eh*PiC in recycling phosphate, which is released during ATP hydrolysis by the action of molecular chaperones. *Eh*ADP/ATP carrier mediates the exchange of cytosolic ATP for mitosomal ADP.

Only two protein sequences were identified in the HMM search of the *E. histolytica* genome: 269.m00084 (E-value of 2.8×10^−94^) and 13.m00296 (E-value of 4.7×10^−5^) (Accession numbers XP_649800 and XP_656350, respectively). The first sequence corresponds to the ADP/ATP carrier [27] and the second to the novel, unannotated protein sequence. Additional analysis of the sequence length and predicted topology support the possibility that 13.m00296 could encode a mitochondrial carrier protein (Figure 1A), one of the most divergent of the carrier protein family. Based on the functional analyses that follow, we labeled the gene as *ehpic* and its translation product as *Eh*PiC.

The 31.7 kDa protein is predicted to have six transmembrane helices, although with the lack of signature motives in the odd-number helices. Only the third helix has a proline residue in the conserved position (Figure 1A). As is the case in fungi, animals and plants, *D. discoideum* has a diverse set of mitochondrial carrier proteins that can be compared in a phylogenetic analyses [31],[32]. When added to the dataset from *D. discoideum*, the *E. histolytica* ADP/ATP carrier clusters together with transporters of adenine nucleotides and coenzyme A, while *Eh*PiC clusters with the P_i_ carrier McfN (Figure 1B). The overall sequence divergence of *Eh*PiC is reflected by a long branch formed in the tree and also by low statistical support. However, a similar tree topology was obtained using different reconstruction methods (Supplementary information Figure S1), which indicated the affinity of *Eh*PiC to McfN. The amino acid residues at three contact sites in the central pore are believed to determine the specificity of the transport channel and their composition can therefore serve for the estimation of the substrate nature [32]. Perhaps due to the high degree of sequence divergence, none of the three sites of *Eh*PiC provided any leads towards the putative pore specificity (Figure 1A). The proposed function of a phosphate carrier in the mitosomes is summarized diagrammatically in Figure 1C.

**Figure 2:**
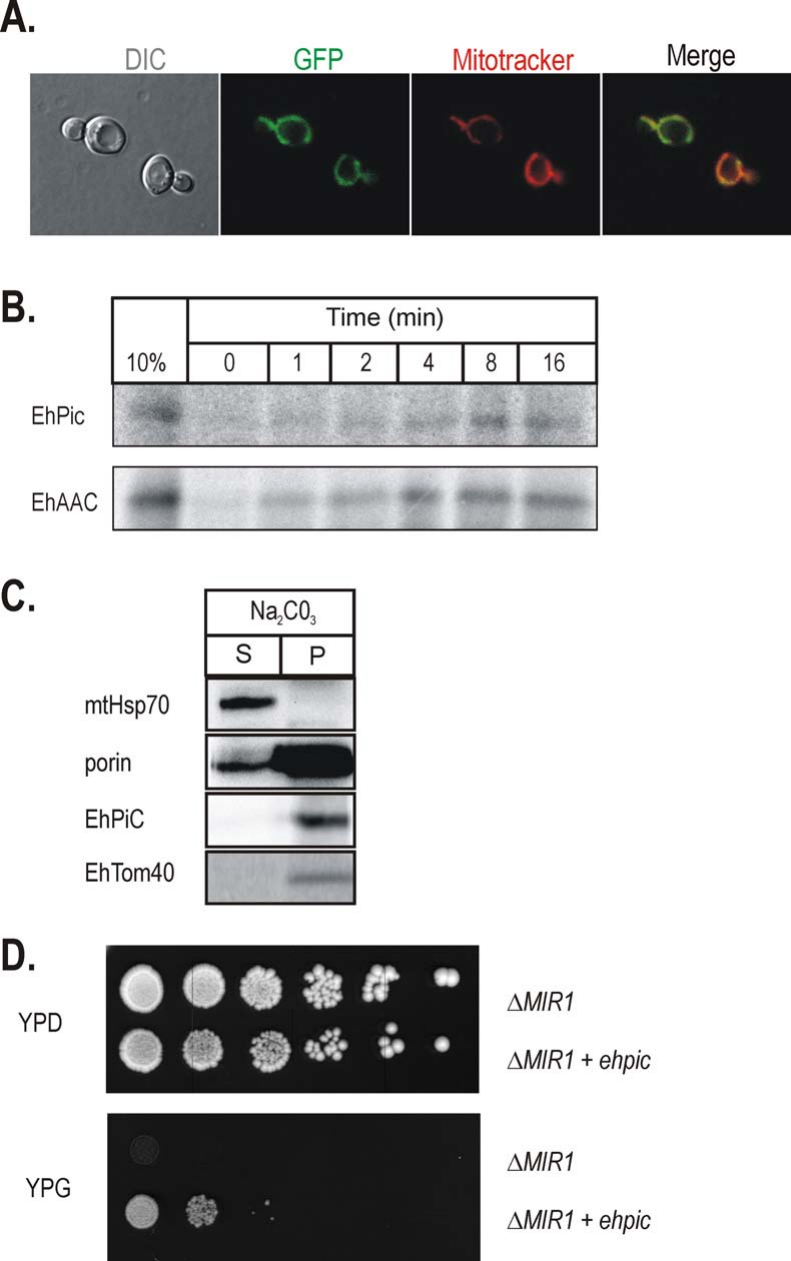
*Eh*PiC is a mitochondrial-type phosphate carrier. (**A**) The C-terminal GFP fusion of *Eh*PiC expressed in yeast cells (green) co-stained with the mitochondria-specific stain Mitotracker red (red). The merged image demonstrates the co-localization of *Eh*PiC with the mitochondrial compartment. DIC – differential contrast (Nomarski). (**B**) ^35^S-labelled *Eh*PiC and *Eh*AAC were incubated with yeast mitochondria, reactions were stopped on ice and the organelles subjected to treatment with proteinase K. 10%–10% of precursor amount used for the import reaction. (**C**) Yeast mitochondria with ^35^S-labelled precursors (after 15 minutes of import) were subjected to treatment with proteinase K and then extracted with sodium carbonate. The immunodecoration of mitochondrial Hsp70 and porin shows the distribution of the typical soluble and the integral membrane proteins, respectively. S-soluble fraction, P-pellet. (**D**) The Δ*mir1* deletion strain [33] was transformed by a plasmid carrying *ehpic* (Δ*mir1*+*ehpic*) or with a plasmid without *ehpic* (Δ*mir1*). Serial dilutions of transformed cells plated on fermentable (YPD) and non-fermentable (YPG) carbon source demonstrate the capability of *Eh*PiC to restore the growth defect of the mutant strain.

To determine whether *Eh*PiC could function as a phosphate carrier protein, we made use of *S. cerevisiae* as a cellular assay system. Fluorescence microscopy showed that *Eh*PiC contains the targeting sequences necessary for the import into mitochondria *in vivo* (Figure 2A). The *Eh*PiC protein can be translated *in vitro* and is imported by mitochondria isolated from *S. cerevisiae* as readily as the *Eh*AAC (Figure 2B). Figure 2C shows that *Eh*PiC behaves as a typical integral membrane protein, exclusively distributed in the pellet fraction after resisting sodium carbonate extraction of mitochondrial membranes. Soluble and peripheral membrane proteins, like mtHsp70, are extracted from membranes by sodium carbonate.

To test if *Eh*PiC functions to transport phosphate, the coding sequence was cloned into a yeast expression vector and transformed into *S.cerevisiae* strain lacking the dominant phosphate carrier (*Δmir1*) [33],[34]. The *Δmir1* mutants grow on glucose containing media but, due to failure to maintain phosphate transport to sustain oxidative phosphorylation, *Δmir1* cells fail to grow on the non-fermentable carbon source glycerol (Figure 2D). *Eh*PiC expression complements the growth defect of these cells, demonstrating that it functions as a phosphate carrier.

### Protein import machinery encoded in the genome of *E. histolytica*

Both *E. histolytica* and the social amoeba *D. discoideum* are amoebozoans, one of the six super-groups of eukaryotes [35],[36]. As a sister clade to the animal and fungal lineages, it was not surprising that the core components of the TOM and TIM machinery, characterized in fungi and animals, have been identified in *D. discoideum* [37]–[40].

Given this conservation in protein import pathways between amoebozoans, fungi and animals, it was highly surprising when the only homologs of the mitochondrial protein import machinery identified in the genome of *E. histolytica* [41] were the chaperones mtHsp70, Cpn60 and Cpn10.

**Figure 3:**
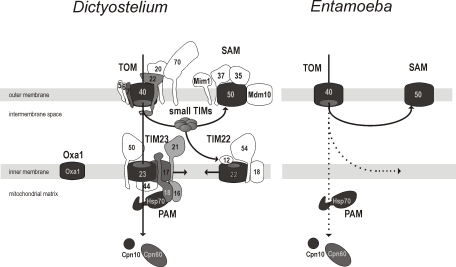
The minimalism of the protein import and assembly pathways in the mitosomes of *E. histolytica.* The schematic representation depicts the complex molecular apparatus in the amoebozoan mitochondria of *D. discoideum* and the mitosome of *E. histolytica* based on hidden Markov model analysis. Of the components present in animal and fungal mitochondria, those shown shaded were found in *D. discoideum*. The panel on the right shows the five components identified in *E. histolytica*. Some of the proteins found in the animals and fungi appear to be missing in Amoebozoa (shown in white). The dashed arrows highlight the unknown translocation pathway across or into the inner mitosomal membrane in *E. histolytica*.

Hidden Markov models were built, representing search tools for 33 mitochondrial components known to participate during the import process either in fungal/animal or plant mitochondria. Each model was built from sequences clearly homologous to the functionally characterized import component. The models were assembled into a library and used to search the *E. histolytica* genome (see Materials and Methods). In parallel we analyzed the genome of *D. discoideum* (Figure 3), which is the most extensively studied species of Amebozoa super-group thus providing a referential dataset for our analysis of the *E. histolytica* genome.

Two sequences 38.m00236 and 137.m00093 (accession numbers XP_655014 and XP_651988) were identified by the Tom40- and Sam50-specific HMMs, respectively. Accordingly, these two open reading frames were named *ehtom40* and *ehsam50* and their putative protein products as *Eh*Tom40 and *Eh*Sam50. The third open reading frame identified in *E. histolytica* corresponds to the previously identified gene encoding for mitochondrial-type Hsp70 [42].

### A core component of the TOM complex: *Eh*Tom40

**Figure 4:**
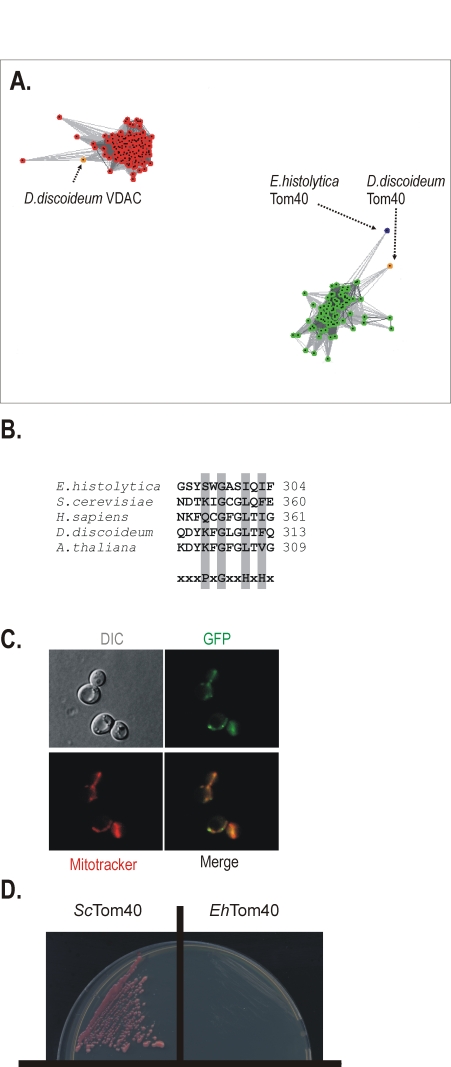
A mitochondrial Tom40 in *Entamoeba histolytica*. (**A**) A representative figure from the CLANS analysis of a data set containing 79 Tom40 sequences (green dots), 137 VDAC (red dots) sequences and the *Eh*Tom40 sequence (blue dot). A P-value threshold of 10^−82^ was used to give complete separation of the VDAC and Tom40 clusters. Tom40 and VDAC sequences from *D. discoideum*, as the closest relative in the dataset, are highlighted (orange). (**B**) The alignment shows the C-terminal β-strand from various Tom40 homologues. The motif for the general import signal for mitochondrial β-barrel proteins is highlighted, P-polar residue, G-glycine residue, H-hydrophobic residue [48]. (**C**) The C-terminal GFP fusion of *Eh*Tom40 in yeast cells (green), the yeast mitochondria stained with Mitotracker red (red). The merged image demonstrates the co-localization of *Eh*Tom40 with the mitochondrial compartment. DIC – differential contrast (Nomarski). (**D**) *S. cerevisiae* Δ *tom40* mutants, carrying a counterselectable *TOM40* gene and expressing either *S. cerevisiae* Tom40 (*Sc*Tom40) or *Eh*Tom40 were plated onto 5-FOA media to select against the covering plasmid. Cells were incubated for 4 days at 30°C and only strain expressing *Sc*Tom40 was viable.

The gene *ehtom40* encodes a protein of 305 amino acids with a theoretical molecular weight of 34.4 kDa, similar in size to the Tom40 from *Encephalizoon cuniculi* [43]. *Eh*Tom40 is expressed in *E. histolytica* with the *ehtom40* mRNA detected in extracts from amoebae by reverse transcription coupled with specific PCR amplification (Supplementary information Figure S2). The reverse BLASTP search with *Eh*Tom40 as a query provided hits to a ‘porin family protein’ from *Arabidopsis thaliana* (NP_175457.1), the Tom40 sequence from *Trimastix pyriformis* (ABW76113.1) and a hypothetical protein from *Leishmania infantum* (XP_001463193.1) representing an unidentified protein predicted to be a β-barrel. Such a structure is typical for Tom40 proteins. The mitochondrial porin 3 superfamily of proteins (Pfam01459) encompasses both the eukaryotic voltage-dependent anion channel (VDAC) proteins and Tom40s [44],[45]. Other mitochondrial β-barrel proteins like Sam50 do not fall within the porin 3 superfamily. Given the similarity of *Eh*Tom40 to β-barrel proteins predicted to be Tom40 (ABW76113.1) and VDAC (NP_175457.1), we performed a CLANS analysis using a sequence set containing 137 VDAC and 79 Tom40 sequences (including *Eh*Tom40). Membrane proteins are often difficult to resolve on phylogenetic trees but CLANS, that graphically depicts homology in large datasets of proteins, has been previously found to be very efficient in the classification of the β-barrel proteins from bacteria [46] and also in the characterization of a Tom40 homologue in *G. intestinalis* [47]. This approach utilizes all-against-all pairwise BLAST, clustering “vertices” (i.e. individual protein sequences) in three-dimensional space using an algorithm which applies a weak repulsive force to each vertex and an attractive force between each pair of vertices. The attractive and repulsive forces are proportional to the BLAST high scoring segment pair score. Figure 4A shows that cluster analysis performed on this superset of porin3 family proteins using a P-value cutoff of 10^−82^ results in the sequences clustering into two distinct groups. The clusters are either exclusively VDAC homologues or Tom40 homologues, corresponding to their independent phylogenies across eukaryotes. The *Eh*Tom40 is positioned on the periphery of Tom40 cluster, clearly separated from the compact cluster of VDAC sequences.

Mitochondrial β-barrel proteins contain a specific mitochondrial targeting signal (the β-signal, PxGxxHxH, where P stands for polar, G for glycine and H for hydrophobic residue) that sits in the last β-strand of the protein [48]. The β-signal is recognized and bound by the SAM complex, which then mediates folding and membrane insertion of the β-barrel. *Eh*Tom40 has a β-signal that strictly follows this rule (Figure 4B). When *Eh*Tom40 is expressed in *S. cerevisiae*, it is specifically targeted to mitochondria, as judged by fluorescence microscopy (Figure 4C). In order to test whether *Eh*Tom40 can functionally substitute for *S. cerevisiae* Tom40 we have transformed a haploid *Δtom40* strain that carries the *S.cerevisiae* TOM40 on a counterselectable plasmid. Upon plating on 5-FOA media, to select against the covering plasmid, no viable transformants were obtained (Figure 4D), while the strain transformed with the homologous *TOM40* remained viable. Apparently the sequence divergence between the fungal and amoebic Tom40 interferes with the correct docking and interaction with other TOM complex subunits and thus does not allow for functional complementation. Therefore we subsequently tested whether *Eh*Tom40 integrates into the native *S. cerevisiae* TOM complex using the *in vitro* import assays. Although the protein accumulated as a high molecular weight species in the mitochondrial membranes (Supplementary information Figure S3), the specific pull-down assay using the antibodies against TOM complex subunits did not recover any of *Eh*Tom40 protein (data not shown).

Given that TOM complex requires numerous binding sites on several distinct subunits to assemble properly, it is not surprising that these experiments failed. It was previously reported that even the dual point mutations in Tom40 of *S.cerevisiae* can result in the disassembly of the native TOM complex [49].

### A core component of the SAM complex: *Eh*Sam50

The cDNA sequence of Sam50 homologue found in *E. histolytica* is deposited at NCBI under accession number XM_646896. It encodes for a protein of 388 amino acids with a theoretical molecular weight of 45.3 kDa. However, when aligned with the corresponding genomic sequence, the presence of an intervening sequence was revealed. The insertion of 72 bp starting at position 802 is limited by GAATGATT and TAG at the 5′- and 3′-ends. These are conserved intron donor and acceptor splice sites identified in *E. histolytica* [50], suggesting that *ehsam50* gene is interrupted by a single intron. According to the molecular weight of the protein when episomally expressed in *E. histolytica* and *S. cerevisiae* the mRNA is likely processed *in vivo* in both cellular systems and provides for *Eh*Sam50 translation.

**Figure 5:**
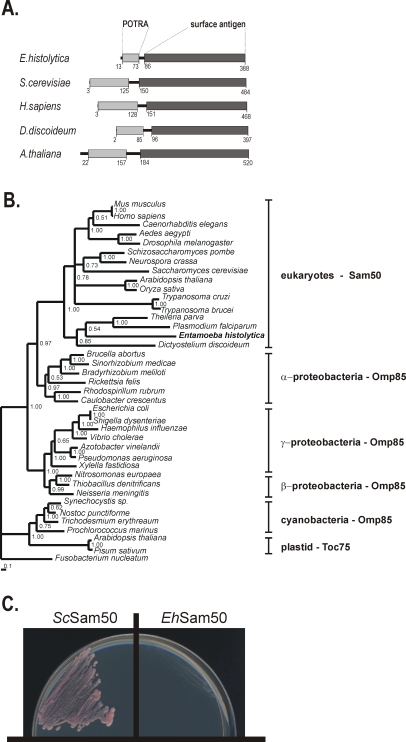
Sam50 in *Entamoeba histolytica*. (**A**) Signature domains present in Sam50: A C-terminal surface antigen domain (dark grey) which corresponds to the membrane embedded β-barrel, and the POTRA domain (light grey) which is exposed to the intermembrane space. Both domains are present in *Eh*Sam50 as determined by Pfam search [45] and HHpred analyses [81]. (**B**) Protein maximum-likelihood phylogenetic tree was derived from a dataset of 407 aligned amino acids from 39 sequences using MrBayes [70]. Numbers at the individual nodes represent MrBayes posterior probabilities. (**C**) *S. cerevisiae* Δ *sam50* mutants, carrying a counterselectable *SAM50* gene and expressing either *S. cerevisiae* Sam50 (*Sc*Sam50) or *Eh*Sam50 were plated onto 5-FOA media to select against the covering plasmid. Cells were incubated for 4 days at 30°C and only strain expressing *Sc*Sam50 was viable.

As shown on phylogenetic reconstruction the protein sequence clusters with other mitochondrial Sam50 sequences [51],[52] with α-proteobacterial Omp85 sequences in a sister clade (Figure 5B) [53], implying that *Eh*Sam50 is of direct mitochondrial origin.

Omp85 proteins contain a C–terminal β-barrel domain (the ‘surface antigen domain’), which is integrated into the outer membrane. A characteristic N-terminal extension consists of five POTRA (polypeptide-transport-associated) domains [54]. In contrast, mitochondrial Sam50 proteins, including *Eh*Sam50, contain a single POTRA domain along a β-barrel domain (Figure 5A). Together with obtained phylogenetic data this provides additional support that *Eh*Sam50 is a genuine mitochondrial homologue, not a bacterial Omp85 acquired by a horizontal gene transfer. While the structural domains are well conserved in *Eh*Sam50, we could not obtain a viable *S.cerevisiae Δsam50* strain expressing the *E. histolytica* protein (Figure 5C). As in the case of Tom40, the primary sequence was likely too divergent to support functional integration of *Eh*Sam50 into SAM complex of *S.cerevisiae*. So far, there is no record in the literature of successful heterologous replacement of SAM complex components, which suggests that tight protein-protein or protein-lipid interactions are involved in the SAM function.

### Localization of proteins in *E. histolytica*

**Figure 6:**
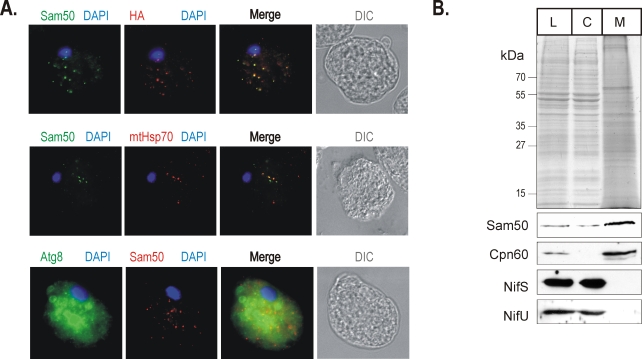
Sam50 is confined to the mitosomal vesicles uniformly distributed through the cytoplasm. (**A**) The upper panel shows fixed *E. histolytica* cells expressing the HA-tagged version of *Eh*Sam50 were probed with α-HA (green) and α -r*Eh*Sam50 (red ) antibody for immnunofluorescent labeling. DIC – differential contrast (Nomarski). In the middle panel HA-tagged *Eh*Sam50 was labeled by α-HA (green) and mtHsp70 with polyclonal α-mtHsp70 (red) raised against *Nyctotherus ovalis* mtHsp70 [57]. The lower panel shows fixed *E. histolytica* cells expressing the HA-tagged version of *Eh*Sam50 were probed with α-Atg8, an autophagosome marker (green) and α-*Eh*Sam50 (red) antibody. (**B**) Cultured *E. histolytica* cells were disrupted and the cell lysate (L), cytosol (C) and mitosome-enriched fraction (M) and probed for NifS, NifU using homologous polyclonal antibodies [41] and Cpn60 using heterologous polyclonal antibody raised against *Giardia intestinalis* Cpn60. *Eh*Sam50 was decorated with α-HA monoclonal antibody.

In order test *Eh*Sam50 *in vivo*, the expression of *Eh*Sam50 (and *Eh*Tom40 and *Eh*PiC) in *E. histolytica* was confirmed by RT-PCR (Supplementary information Figure S2). As an experimental system, *E. histolytica* is challenging. In order to detect whether *Eh*Sam50, *Eh*Tom40 and *Eh*PiC are localized to a subcellular compartment, we set about both raising antisera to the three proteins and creating HA-tagged versions of the proteins for expression in *E. histolytica*. Neither strategy allowed localization of *Eh*PiC or *Eh*Tom40 in *E. histolytica* (recent data of Mi-Ichi et al. support the mitosomal distribution of *Eh*Tom40 [22]). However, an α-*Eh*Sam50 specific serum was raised and an *E. histolytica* strain expressing *Eh*Sam50 with a C-terminal HA-tag was cultivated. Fixed *E. histolytica* cells were probed with α-HA and with either α-*Eh*Sam50, α-mtHsp70 or α-Atg8 antibody and immunofluorescent labeling determined. Atg8 is a marker of autophagocytic vesicles, which might be of similar morphology to mitosomes [55]. *Eh*Sam50 was found in vesicles with a size and overall pattern similar to the cpn60-specific labeling by León-Avila and Tovar [56]. The co-distribution of HA- and *Eh*Sam50- specific signal on the merged image shows that the antibodies specifically label the same cellular compartment (Figure 6A), distinct from the Atg8-specific labeling of autophagosomes [55]. More importantly, using a α-mtHsp70 raised against mitochondrial Hsp70 from *Neocallimastix patriciarum* [57] the specific co-localization with *Eh*Sam50 was found. This data strongly supports the mitosomal distribution of *Eh*Sam50 together with mitosomal Hsp70 [42].

To further test the mitosomal distribution of the protein import machinery in *E. histolytica*, the cell fractions were probed for the mitosomal and the cytosolic marker proteins (Figure 6B). While our data suggested the cytosolic distribution of NifU and NifS proteins as found in the analysis of Mi-Ichi et al. [22], *Eh*Sam50 and mitosomal Cpn60 were distributed in the high-speed pellet fraction containing mitosomes.

## Discussion

Hidden Markov models can be used for a highly sensitive search of sequence databases [58], and discovered three new mitosomal proteins in *E. histolytica*. Tom40 provides a channel for the import of mitochondrial proteins including Sam50 and Tom40 itself, whereas Sam50 is necessary for assembly of the Tom40 channel (and Sam50 itself). The characterization of homologs of Sam50 and Tom40 in *E. histolytica* suggests that mitosomes have a functional outer membrane as also recently shown by Maralikova et al. [21]. It is now clear, even with so few mitosomal proteins identified in *E. histolytica*, that we have in hand marker proteins for the outer membrane (Sam50, Tom40), the inner membrane (ADP/ATP carrier, PiC, sodium/sulfate symporter) and the mitosomal matrix (e.g. molecular chaperones, rubrerythrin, ATP sulfurylase).

The inner membrane of all mitochondria known so far is populated by the proteins from Tim17/22/23 family. These proteins contain four membrane spanning helices, by which they build up the core channels of the translocase of the inner membrane (TIM). In animals fungi, plants and diverse protists groups these proteins are present in three different forms as Tim17, Tim22 and Tim23, which play distinct roles in the import of matrix and/or inner membrane proteins. Some eukaryotes, notably trypanosomes and microsporidians, appear to make use of just a single Tim17/22/23 protein [13],[59], which might functionally substitute for the three distinct forms as a “jack of all trades”. Despite the high degree of similarity among the Tims, from these various eukaryotes including *D. discoideum*, the HMM models of the Tim17/22/23 family of proteins did not reveal any homologous sequences in *E. histolytica*. This could either mean that the putative *E. histolytica* Tims have diverged in sequence to such an extent as to be beyond the sensitivity of our HMM models, or that *E. histolytica* lacks any Tim17/22/23-related protein due to the overall mitochondrial reduction. Failure to detect a Tim17 family protein in mitosomes of *G. intestinalis* argues for such a common reductive step in mitochondrial adaptation [10],[60]. An interesting question is raised about the mechanism of carrier protein assembly in the mitosomal membranes of *E. histolytica* as these proteins normally require functional TIM22 translocase.

Owing to its extreme lifestyle, *E. histolytica* is believed to depend solely on cytosolic production of ATP through glycolysis [61]. The ADP/ATP carrier in *E. histolytica* mitosomes provides for retrograde transport of ATP into the mitosomal matrix, enabling the ATP-dependent activity of Hsp70 and Cpn60 as well as ATP sulfurylase and APS kinase [17],[22],[28],[29]. Our finding of the phosphate carrier *Eh*PiC completes this metabolic shunt.

### Targeting of the proteins into *E. histolytica* mitosomes

Most of soluble mitochondrial proteins contain cleavable N-terminal targeting sequence that usually forms an amphipathic helix, which is recognized by the receptor on the outer mitochondrial membrane and upon the translocation into mitochondrial matrix cleaved off by a matrix processing peptidase (MPP) [62]. The robust bioinformatic search for more proteins with putative mitosomal targeting sequences in *E. histolytica* is hindered by very poor training dataset available to date (Supplementary information Figure S4) [22]. However, in our additional HMM search for a putative MPP homologue we identified single candidate protein from M16 peptidase family and we are currently characterizing this protein. Although MPP is a heterodimer of two different but evolutionary related subunits, a single subunit minimalist version of the enzyme was reported recently in the mitosomes of *G. intestinalis* [60]. Given that mitosomes of *E. histolytica* represent even more reduced mitochondria one can anticipate analogous reduction occurring herein.

### Protein import into amoebic mitochondria - a window into mitochondrial evolution

Phylogenetic reconstructions and the presence of flagellate cells in the life-cycles of some Amoebozoans suggested an affiliation of this supergroup with animals and fungi [36], so that studies on fundamental pathways in the Amoebozoa provide important additional information on the cell biology of the earliest animals and fungi.

Genome sequence analysis of the highly-studied amoebozoan, *D. discoideum*, revealed the presence of 14 different components taking part in the mitochondrial protein import in animals and fungi (see Figure 3). This analysis suggests that all four major membrane complexes: SAM, TOM, TIM22 and TIM23 are present in *D. dictyostelium*, as is the intermembrane space-located small TIM chaperones that shuttle substrates to the SAM or TIM22 complexes. The presence of these various complexes in *D. discoideum* is consistent with them having been installed in the earliest stages of eukaryote evolution. The components that are missing in *D. discoideum* are those that appear to be specific for fungi and animals, as has been previously discussed for receptor subunits Tom20 [63] and Tom70 [64]. That so many TOM and TIM components are missing from *E. histolytica*, is strong evidence for a secondary gene loss having occurred, as part of the reductive evolution impacting on the mitosomal organelle. The anaerobic lifestyle, combined with parasitism, of *E. histolytica* likely selected for a minimalist mitochondrial set up and the enormous reduction of the import machinery. This likely reflects the even more dramatic reduction of the overall mitochondrial metabolism. The anaerobic, free-living amoeba *Mastigamoeba balamuthi* looks to have traveled part way the same direction: 21 putative mitochondrial proteins have been identified in the limited EST dataset, while no TOM, TIM and SAM components were found so far [65].

The recent data on the function of *E. histolytica* mitosome have revealed remarkable divergence of the processes occurring within this mysterious mitochondrion. It now seems that while these organelles have lost vast majority of typical mitochondrial functions, they have accommodated several new unexpected roles not seen in other mitochondria [21],[22]. In this paper, we uncovered the essentials of the protein import into these organelles and it is very likely that the further research on the function and the biogenesis of *E. histolytica* mitosome will bring more of the unexpected.

## Materials and Methods

### Sequence search and analysis

The Hidden Markov models describing 33 mitochondrial components were constructed and compiled into a HMM library from manually prepared families of sequences (Oxa1, Tim10, Tim44, Tom20opistho, Tom20plants, Tom70, Pam16, Tim13, Tim50, Tom22opistho, Tom22plants, Hsp70, Pam18, Tim17, Tim54, Sam35, Tim18, Tim8, Tom40, Mdm10, Sam37, Tim21, Tim9, Tom5, Metaxin1, Sam50, Tim22, Tim9+10, Tom6, Metaxin2, Tim23, and Tom7). The HMM library was used to scan the genomes of *E. histolytica* (http://www.tigr.org/tdb/e2k1/eha1/) and *D. discoideum* (NCBI). In addition, a hidden Markov model based on 34 manually compiled MCP sequences was built and used to scan the two genomes. The program HMMER 2.3.2 was used in all calculations [66], and the search results were extracted with the programs prepared in-house.

The homology modeling of the mitochondrial carrier protein was performed with SwissModel at http://swissmodel.expasy.org//SWISS-MODEL.html [67]. The structure of bovine ATP/ADP carrier (PDB ID 2C3E) was used as a template [30]. The sequences were aligned using ClustalX [68] and edited manually in BioEdit (http://www.mbio.ncsu.edu/BioEdit/bioedit.html).

For the neighbor joining analysis of mitochondrial carrier proteins amino acid sequences were aligned and the resulting alignment edited manually into a dataset of 33 sequences of 190 amino acid residues. SplitsTree4 [69] software was used to calculate the bootstrapping of 500 runs and to combine the results into a Neighbor-Net.

Protein maximum-likelihood phylogenetic tree of Omp85/Toc75/Sam50 proteins was derived from a dataset of 407 aligned amino acids from 39 sequences. The tree was obtained using the program MrBayes under the JTT substitution matrix with amino acid frequencies estimated from the dataset [70]. Site rate variation was modeled under a discrete approximation to the Γ distribution (one invariable and four variable rate categories). Four Monte Carlo Markov Chains, each with 2,000,000 generations, were performed with trees sampled every 100 generations. For compilation of Bayesian consensus topologies, a “burn-in” of 500 trees was used.

CLANS version 2 October 9, 2006 was obtained from http://bioinfoserver.rsbs.anu.edu.au/programs/clans/. The *Eh*Tom40 sequence was added to a sequence set of 137 VDAC and 79 Tom40 sequences); the sequence set was derived from both the results of BLAST and HMM searches using VDAC and Tom40 sequences. Cluster analysis was performed with a P-value cutoff (10^−82^) sufficient to observe complete separation of the VDAC and Tom40 sequence clusters. As the method is non-deterministic, the analysis was run until stable clusters formed (in excess of 200 iterations). Multiple runs were performed to ensure that the observed clusters formed using different starting positions for the sequences.

### *Entamoeba histolytica* culture and preparation of RNA

Trophozoites of the *E. histolytica* isolate HM-1:IMSS were cultured axenically in TYI-S-33 medium in plastic tissue culture flasks [71]. For further experiments 1×10^6^ trophozoites were cultivated for 24 h in 75 ml culture flasks. The trophozoites were then harvested after being chilled on ice for 5 min and sedimented at 430×g at 4°C for 5 min. The resulting pellet was washed once in phosphate-buffered saline pH 7.2 and once in 20 mM MOPS pH 7.2. The cell pellet was resuspended in 2 ml of 20 mM MOPS pH 7.2 with protease inhibitors and passed 6 times through a 25 G needle until most cell were broken. The cell lysate was diluted with 25 ml of 250 mM Sucrose, 20 mM MOPS pH 7.2 and spun down twice at 650×g for 10 min, resulting supernatant was spun down at 2,850×g for 10 min. The final high-speed pellet representing the mitosomal fraction was obtained after centrifugation at 100,000×g for 30 min. The high-speed supernatant corresponded to the cytosolic fraction.

For total RNA isolation 1×10^6^ *E. histolytica* trophozoites were cultivated in 75 ml culture flasks for 24 h. The cells were harvested via chilling on ice for 5 min and sedimented at 200×g for 5 min at 4°C. The cell pellet was washed twice with PBS. The trophozoites were treated with TRIZOL reagent (Invitrogen) following the manufacturer's instructions. Extracted RNA was purified using the RNeasy mini kit (Qiagen) without β-mercaptoethanol and DNA was digested with DNase (Qiagen). cDNA synthesis was accomplished with SuperScriptIII Reverse Transcriptase (Invitrogen). In a final volume of 20 µl, 1 µg of RNase-free and DNase-treated total RNA was mixed with 5×First-Strand buffer, 500 µM dNTPs, 500 nM OdT-T71 (5′-GAG AGA GGA TCC AAG TAC TAA TAC GAC TCA CTA TAG GGA GAT24), 2 mM DTT, 40 U RnaseOut (Invitrogen) and SuperScriptIII (200 U/µl). cDNA was synthesised for 1 h at 42°C.

### Yeast culture and cell fractionation

*Saccharomyces cerevisiae* strain W303a was grown in rich medium or selective medium as previously described [72]. The *Δmir1* strain was a gift from Dr. Geneviève Dujardin (Centre de Génétique Moléculaire, CNRS, Paris, France) [33]. For the preparation of mitochondria for the *in vitro* study *S. cerevisiae* strain W303a was grown in lactate media at 25°C. The mitochondria were isolated by differential centrifugation as described previously [73]. For the growth assays the cells were grown to a mid-logarithmic phase in a complete media, diluted into OD_600_ = 0.2, spotted in the series of fivefold dilutions on the plates and incubated at 30°C for 3–6 days.

### Cloning and expression of *ehpic*, *ehtom40* and *ehsam50*

For GFP-tagging, the open reading frames were amplified by PCR using *E. histolytica* genomic DNA as template and primers containing 5′*EcoR*I and 3′*BamH*I or *Bgl*II sites (see Table S1) and cloned into p416MET25 vector [74]. To create the C-terminal HA-tag fusions the ORFs were cloned into a pYX143 vector with the use of 5′*EcoR*I and 3′ *Mlu*I restriction sites. For the expression of *ehsam50* in *E. histolytica*, the ORF was amplified from pYX143 with the C-terminal HA-tag using the 5′*Kpn*I and 3′ *Bgl*II restriction sites. The plasmid (pNC) used for transfection is a derivative of the expression vector EhNEO/CAT. The plasmid contains the neomycin phosphotransferase-coding sequence flanked by 480 bp of the 5′-untranslated sequence and 600 bp of the 3′-untranslated sequence of an *E. histolytica* actin gene. Transfections were performed by electroporation as described previously [75]. Drug selection started 48 h after transfection, using 10 µg/ml of the neomycin analogue G418. Two weeks later, the G418 concentration was increased to 50 µg/ml.

For the production of *Eh*Sam50-derived antigen the first 300 bp of *ehsam50* were amplified by PCR and ligated into pET23a vector (Novagen) using 5′*Nde*I and 3′ *Xho*I restriction sites. *E. coli* strain BL21 (DE3) was used to produce the recombinant protein with C-terminal six histidine tag. The protein was purified to homogeneity under denaturing condition (8 M urea) on NTA-nickel column (Qiagen). For generation of polyclonal antibodies 100 µg recombinant *Eh*Sam50 (r*Eh*Sam50) was injected into a mouse, followed by two further injections.

### *In vitro* protein import

Mitochondria were prepared according to the method of Daum et al [73]. For *in vitro* transcription the genes were amplified by PCR with the forward primers containing the SP6 promoter followed by a Kozak's sequence (*ehpic*, *ehtom40*, *ehsam50*) or cloned into pSP73 vector (*ehAAC*), which was linearized at a unique site downstream of the gene. [76],[77]. In vitro imports were assayed according to Gabriel and Pfanner [78].

### Immunofluorescence microscopy and western blot analysis

*E. histolytica* cells were analyzed with α-*Eh*Sam50 antiserum, with an antiserum raised against the *E. histolytica* autophagosome marker Atg8 (autophagy related gene 8, a gift from Dr. Tomoyoshi Nozaki, National Institute of Infectious Diseases, Tokyo, Japan) or with a mouse monoclonal anti-HA antibody. Cells were fixed at room temperature for 30 min in PBS containing 3% paraformaldehyde and subsequently permeabilized with 0.05% saponin (PBSS). Samples were incubated at room temperature for 1 h with antisera against *Eh*Sam50 (1∶250 dilution), against Atg8 (1∶500 dilution) or against the HA-tag (1∶200, Roche). Secondary antibodies were Alexa-594 coupled α-mouse, Alexa-488 coupled α-rabbit, and Alexa-594 coupled α-mouse antibodies. Subsequently, cells were mounted on glass slides and examined under 6300× magnification. For deconvolution microscopy images of selected cells were captured with a 63× oil immersion lens in a UV equipped Leica DM RB microscope with 0.2-µm-diameter step Z-sections. Deconvoluted Z sections were examined for colocalisation of Atg8 and *Eh*Sam50 staining with the Openlab 4.0.4 program. Adobe Photoshop 7.0.5 was used for additional processing of the images. *S. cerevisiae* were analyzed as previously described (Beilharz et al. 2003). ImageJ software was used for additional image processing (http://rsbweb.nih.gov/ij/). In the western blot analysis cell fractions were probed with the mouse monoclonal anti-HA antibody (1∶500), rabbit polyclonal antibodies raised against *Giardia intestinalis* Cpn60 (1∶1000) (a kind gift from Dr. Robert Hirt, Newcastle University, UK), *E. histolytica* NifS and NifU (both 1∶1000) (a kind gift from Dr.Tomoyoshi Nozaki [26]).

The distribution of *Eh*PiC, *Eh*Tom40 and *Eh*Sam50 in *S. cerevisiae* mitochondria extracted in fresh 100mM Na_2_CO_3_ was done as previously described [79].

## Supporting Information

Figure S1 Neighbor joining tree of 31 mitochondrial carrier proteins from *D. discoideum* with ADP/ATP carrier and PiC carrier from *E. histolytica* constructed by SplitsTree4 [86]. The carriers cluster according to their substrate specificity. The putative substrates of *D. discoideum* carriers [87] are indicated as follows: *Carn/Orn* - carnitine or ornithine, *Asp/Glu* - aspartate/glutamate, *Oglu* - 2-oxoglutarate, *Dic/Tric* - dicarboxylate/tricarboxylate, *Pi* - phosphate, *PyrNucl* - pyrimidine NTP/NMP, *perox ATP* - peroxisomal ATP carrier, *H* ^+^ *FA* - H^+^ fatty acid, *CoA* - coenzyme A, *dNucl* - deoxynucleotide, *AA-Mn* - amino acid (Mn^2+^), *Fe* - iron (mitoferrin).
(0.12 MB PDF)

Figure S2 The products of specific RT are shown: 1 - *ehpic*, 2 - *ehtom40*, 3 - *ehsam50*. The marker is in base pairs.
(0.05 MB PDF)

Figure S3 ^35^S-labelled *Eh*Tom40 was incubated with yeast mitochondria, solubilized with 1% digitonin and the samples resolved on BN-PAGE. The time-dependent formation of high-molecular weight complexes in the mitochondrial membranes is demonstrated. The large white arrow points to disappearance of the monomeric form of *Eh*Tom40 in the reaction, small black arrows highlight the formations of the of high-molecular weight complexes.
(0.05 MB PDF)

Figure S4 The protein sequence alignments of all three known soluble mitosomal matrix proteins Cpn10, Cpn60, Hsp70 with their bacterial and eukaryotic counterparts. The presence of extremely short N-terminal targeting sequence does not allow for creation of a prediction algorithm.
(0.07 MB PDF)

TableS1 Primers used in the study.
(0.03 MB PDF)
