# Supplementary material for: The Essentials of Protein Import in the Degenerate Mitochondrion of Entamoeba histolytica
Source: PLoS Pathog. 2010 Mar 19;6(3):e1000812. doi: 10.1371/journal.ppat.1000812 (PMC2841616; doi:10.1371/journal.ppat.1000812)
Supplement: Figure S1 — Neighbor joining tree of 31 mitochondrial carrier proteins from D. discoideum with ADP/ATP carrier and PiC carrier from E. histolytica constructed by SplitsTree4 [86]. The carriers cluster according to their substrate specificity. The putative substrates of D. discoideum carriers [87] are indicated as follows: Carn/Orn - carnitine or ornithine, Asp/Glu - aspartate/glutamate, Oglu - 2-oxoglutarate, Dic/Tric - dicarboxylate/tricarboxylate, Pi - phosphate, PyrNucl - pyrimidine NTP/NMP, perox ATP - peroxisomal ATP carrier, H + FA - H+ fatty acid, CoA - coenzyme A, dNucl - deoxynucleotide, AA-Mn - amino acid (Mn2+), Fe - iron (mitoferrin). (0.12 MB PDF) [file ppat.1000812.s001.pdf]

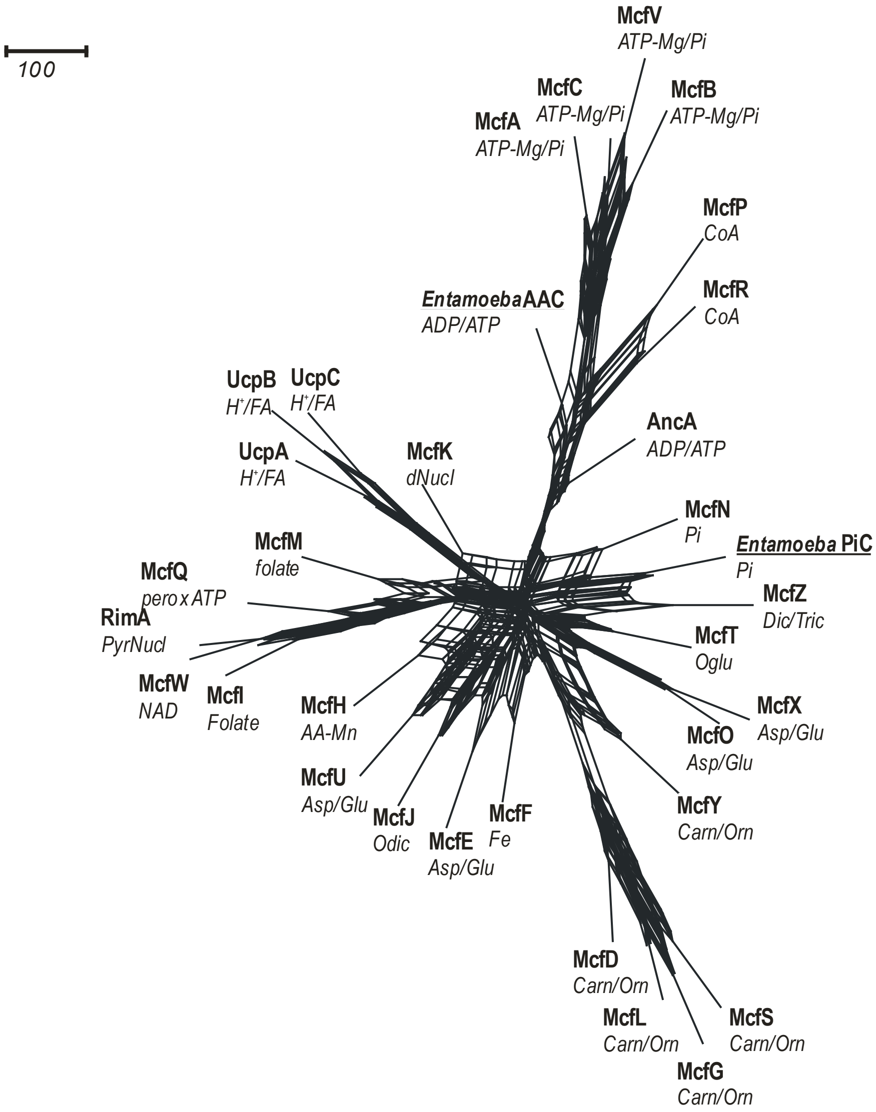

Supporting Figure 1

Neighbor joining tree of 31 mitochondrial carrier proteins from *D. discoideum* with ADP/ATP carrier and PiC carrier from *E. histolytica* constructed by SplitsTree4 [86]. The carriers cluster according to their substrate specificity. The putative substrates of *D. discoideum* carriers [87] are indicated as follows: *Carn/Orn* – carnitine or ornithine, *Asp/Glu* – aspartate/glutamate, *Oglu* – 2-oxoglutarate, *Dic/Tric* – dicarboxylate/tricarboxylate, *Pi* – phosphate, *PyrNucl* – pyrimidine NTP/NMP, *perox*

ATP – peroxisomal ATP carrier, *H^+ FA* – H^+ fatty acid, *CoA* – coenzyme A, *dNucl* – deoxynucleotide, *AA-Mn* – amino acid (Mn^{2+}), *Fe* – iron (mitoferrin).
