# Supplementary material for: The Essentials of Protein Import in the Degenerate Mitochondrion of Entamoeba histolytica
Source: PLoS Pathog. 2010 Mar 19;6(3):e1000812. doi: 10.1371/journal.ppat.1000812 (PMC2841616; doi:10.1371/journal.ppat.1000812)
Supplement: Figure S2 — The products of specific RT are shown: 1 - ehpic, 2 - ehtom40, 3 - ehsam50. The marker is in base pairs. (0.05 MB PDF) [file ppat.1000812.s002.pdf]

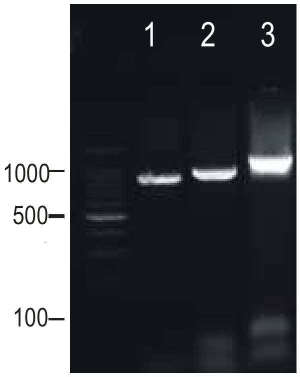

Supporting Figure 2

The products of specific RT are shown: 1 – *ehpic*, 2 - *ehtom40*, 3 – *ehsam50.* The marker is in base pairs.
