# Supplementary material for: The Essentials of Protein Import in the Degenerate Mitochondrion of Entamoeba histolytica
Source: PLoS Pathog. 2010 Mar 19;6(3):e1000812. doi: 10.1371/journal.ppat.1000812 (PMC2841616; doi:10.1371/journal.ppat.1000812)
Supplement: Figure S3 — 35S-labelled EhTom40 was incubated with yeast mitochondria, solubilized with 1% digitonin and the samples resolved on BN-PAGE. The time-dependent formation of high-molecular weight complexes in the mitochondrial membranes is demonstrated. The large white arrow points to disappearance of the monomeric form of EhTom40 in the reaction, small black arrows highlight the formations of the of high-molecular weight complexes. (0.05 MB PDF) [file ppat.1000812.s003.pdf]

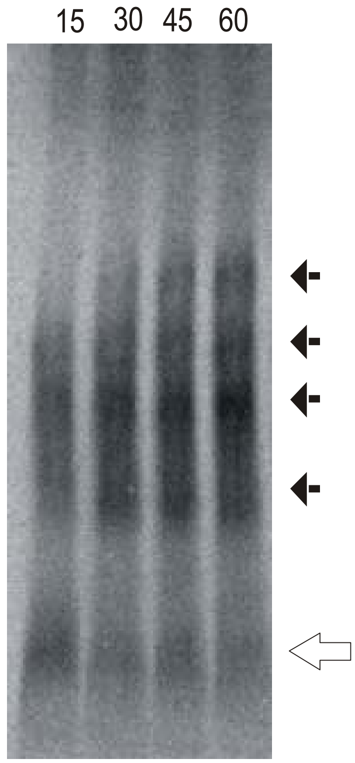

Supporting Figure 3

^{35}S-labelled *Eh*Tom40 was incubated with yeast mitochondria, solubilized with 1% digitonin and the samples resolved on BN-PAGE. The time-dependent formation of high-molecular weight complexes in the mitochondrial membranes is demonstrated. The large white arrow points to disappearance of the monomeric form of *Eh*Tom40 in the reaction, small black arrows highlight the formations of the of high-molecular weight complexes
