# Supplementary material for: The Essentials of Protein Import in the Degenerate Mitochondrion of Entamoeba histolytica
Source: PLoS Pathog. 2010 Mar 19;6(3):e1000812. doi: 10.1371/journal.ppat.1000812 (PMC2841616; doi:10.1371/journal.ppat.1000812)
Supplement: Figure S4 — The protein sequence alignments of all three known soluble mitosomal matrix proteins Cpn10, Cpn60, Hsp70 with their bacterial and eukaryotic counterparts. The presence of extremely short N-terminal targeting sequence does not allow for creation of a prediction algorithm. (0.07 MB PDF) [file ppat.1000812.s004.pdf]

(A) Cpn10

```
E.coli GroES                   MNIRPLHDRVIVKRKEVETKSAGGIVLTGSAA 32
E.histolytica Cpn10             MAKIKPTGDMVLVQHYTTQTVN--GIILAEQKN 31
H.sapiens Cpn10              MAGQAFRKFLPLFDRVLVERSAAETVTKGGIMLPEKSQ 38
S.cerevisiae Cpn10         MSTLLKSAKSIVPLMDRVLVQRIKAQAKTASGLYLPEKNV 40
```

(B) Cpn60

```
E.coli GroEL                                       MAAKDVKFGNDARVKM 16
E.histolytica Cpn60                         MLSSSSHYNGKLLSLNIDCRENV 23
H.sapiens Cpn60            MLRLPTVFRQMRPVSRVLAPHLTRAYAKDVKFGADARALM 40
S.cerevisiae Cpn60            MLRSSVVRSRATLRPLLRRAYSSHKELKFGVEGRASL 37
```

(C) Hsp70

```
E.coli DnaK
E.histolytica Hsp70
H.sapiens Hsp70            MISASRAAAARLVGAAASRGPTAARHQDSWNGLSHEAFRL 40
S.cerevisiae Ssc1                                   MLAAKNILNRSSLSSSF 17

E.coli DnaK                           MGKIIGIDLGTTNSCVAIMDGTTPRVLEN 29
E.histolytica Hsp70             MFVSQPARSTCIGIDLGTTNSCMCVFDKTTPRILEN 36
H.sapiens Hsp70            VSRRDYASEAIKGAVVGIDLGTTNSCVAVMEGKQAKVLEN 80
S.cerevisiae Ssc1          RIATRLQSTKVQGSVIGIDLGTTNSAVAIMEGKVPKIIEN 57
```

Supporting Figure 4

The protein sequence alignments of all three known soluble mitosomal matrix proteins Cpn10, Cpn60, Hsp70 with their bacterial and eukaryotic counterparts. The presence of extremely short N-terminal targeting sequence does not allow for creation of a prediction algorithm.
