# Supplementary material for: The Essentials of Protein Import in the Degenerate Mitochondrion of Entamoeba histolytica
Source: PLoS Pathog. 2010 Mar 19;6(3):e1000812. doi: 10.1371/journal.ppat.1000812 (PMC2841616; doi:10.1371/journal.ppat.1000812)
Supplement: TableS1 — Primers used in the study. (0.03 MB PDF) [file ppat.1000812.s005.pdf]

EhSam50KpnIF	5'- GCTAGGTACCATGTATAACAAAGTA - 3'
HASTOPBglIIR	5'- CTAGAGATCTTCATGCGTAGTCAGG- 3'
EhPiCSP6KozakF	5' – GGATTTAGGTGACACTATAGAATACCGGTGGTACCG CCACCATGGATGGAGTTCAAAGATCCT- 3'
Tim23STOPXhoIR	5'- GTCCTCGAGTTAAAAAAGCCAGAAATG- 3'
EhSam50SP6KozakF	5'- GGATTTAGGTGACACTATAGAATACCGGTGGTACCG CCACCATGGATGTATAACAAAGTATTG- 3'
EhSam50BglIISTOPR	5'- GACAGATCTTTAAAATGAAGTGGTTAT- 3'
EhTom40FSP6KozakF	5'- GGATTTAGGTGACACTATAGAATACCGGTGGTACCG CCACCATGGATGACAAAAGGTACAAAT- 3'
EhTom40BglIISTOPR	5'- GACAGATCTTTAACGAAAGATTTGAAT- 3'
EhAACXhoIF	5'- CAGCTCGAGATGATACAAGGTATGACT- 3'
EhAACBglIISTOPR	5'- GACAGATCTTTACAATTTAAAGAACTT- 3'
EhSam50NdeIF	5'- ATCCATATGTATAACAAAGTATTG- 3'
EhSam50XhoIR	5'- GTCCTCGAGTCCACTTTCTAAATAATT- 3'

Table S1

Primers used in the study.
